# Type 2 diabetes and the cardio–renal–metabolic syndrome: The evolving roles of GLP-1 and dual GLP-1/GIP receptor agonists

**DOI:** 10.17925/EE.2026.22.2.3

**Published:** 2026-08-25

**Authors:** Clipper F Young, Namrita George, Alexander Olson, Diana Isaacs

**Affiliations:** 1. Touro University California College of Osteopathic Medicine, Vallejo, CA, USA; 2. Kirk Kerkorian School of Medicine at the University of Nevada, Las Vegas, NV, USA; 3. Cleveland Clinic Endocrinology & Metabolism Institute, Cleveland, OH, USA

**Keywords:** Cardio-renal-metabolic syndrome, complication reduction and management, diabetes mellitus, type 2, glucagon-like peptide-1 (GLP-1) and glucose-dependent insulinotropic polypeptide (GIP) dual receptor agonist (RA), glucagon-like peptide-1 receptor agonists (GLP-1 RAs), guideline-driven care

## Abstract

The impact of type 2 diabetes (T2D) extends beyond mere management of blood glucose; it also encompasses related cardiovascular, renal and metabolic issues. This interconnected framework, known as the cardio-renal-metabolic (CRM) syndrome, calls for a shift in therapeutic approach. While traditional treatments have made progress focusing on glucose management, a residual risk still exists for conditions such as atherosclerotic cardiovascular disease, chronic kidney disease, heart failure and obesity. The 2025 American Diabetes Association Standards of Care now emphasize treatment algorithms based on risk and complication management, prioritizing comorbidities associated with the CRM syndrome rather than solely focusing on glucose management. This article consolidates current evidence, mechanistic insights and clinical trial findings that support the use of glucagon-like peptide-1 receptor agonists (GLP-1 RAs) and the dual GLP-1/glucose-dependent insulinotropic polypeptide (GIP) receptor agonist to reduce CRM risk. We examine key cardiovascular and renal outcomes trials, as well as metabolic-related trials, such as weight loss and the management of metabolic dysfunction-associated steatotic liver disease (MASLD) and metabolic dysfunction-associated steatohepatitis (MASH), and their alignment with guideline-directed care. In addition to efficacy and safety, we also focus on addressing disparities in access and overcoming implementation challenges, such as low use among specific subpopulations. Lastly, we explore the future of incretin-based therapies, including new triple agonists and ongoing research. Prompt and equitable adoption of GLP-1-based therapeutic options could improve the standard of care for T2D by addressing the entire range of CRM syndrome.

In response to the increasing prevalence of obesity, the consumption of high-calorie and high-sugar foods and an unprecedented aging population, type 2 diabetes (T2D) continues to rise globally. The International Diabetes Federation reported that 589 million individuals aged 20 to 79 years had been diagnosed with diabetes by the end of 2024.^1^ The world prevalence of diabetes is estimated to increase by 45% to 853 million in this age group by 2050.^1^ Recent data from the European Union indicated that the number of adults with diabetes nearly doubled between 2000 and 2019, from 16.8 million to 32.3 million.^2^

The treatment approach for T2D has been expanded beyond blood glucose management. The comprehensive treatment plan addresses associated cardiovascular, renal and metabolic concerns. This interconnected framework, referred to as the cardio–renal–metabolic (CRM) syndrome, necessitates a transformative shift in therapeutic approaches. Although conventional glucose-lowering therapies have improved glycemic management, a residual risk persists for atherosclerotic cardiovascular disease (ASCVD), chronic kidney disease (CKD), heart failure (HF) and obesity-related complications – reflecting the multifactorial pathophysiology of cardiometabolic disease beyond hyperglycemia alone. These conditions collectively contribute to morbidity, mortality and healthcare expenses for individuals with diabetes globally.^3^ ASCVD remains the leading cause of death among adults with diabetes, while CKD significantly contributes to reduced quality of life and premature mortality.^4,5^

In 2020, the US Food and Drug Administration (FDA) issued a draft guidance for considering broader evaluations beyond cardiovascular outcomes trials (CVOTs) when conducting safety trials of medications used to treat T2D.^6^ Technological advancements and a comprehensive understanding of T2D have led to the potential for individuals with T2D and concomitant CRM to derive significant benefits from glucagon-like peptide-1 receptor agonists (GLP-1 RAs) and dual GLP-1/glucose-dependent insulinotropic polypeptide (GIP) receptor agonists. These medications effectively manage blood glucose levels and mitigate the burden of CRM syndrome. Given the robust data on cardiovascular and renal outcomes, these agents have garnered significant interest for the management of type 2 diabetes and cardiometabolic conditions and are currently under investigation for use in people with MASLD and MASH.^7,8^

This article consolidates current evidence, mechanistic insights and clinical trial findings supporting the use of GLP-1 RAs and the dual GLP-1/GIP RA to mitigate CRM risk in individuals with T2D. Additionally, this article highlights how the 2026 American Diabetes Association (ADA) Standards of Care (SOC) in Diabetes reflect this paradigm shift, emphasizing cardiovascular and renal protection, as well as weight management, as priorities, rather than solely focusing on glucose management.

## CRM syndrome in T2D

CRM syndrome is a complex condition arising from the intersection of T2D, obesity, kidney disease and cardiovascular disease. Excess adipose tissue significantly increases the risk of developing T2D. A hallmark manifestation of this metabolic condition is hyperglycemia, which directly affects the renal system.^9^ Chronic hyperglycemia initially causes glomerular hyperfiltration driven by the activation of the renin–angiotensin–aldosterone (RAA) system in response to increased sodium resorption. Over time, hyperfiltration leads to renal tubular cell atrophy, glomerular injury and proteinuria. Angiotensin II and aldosterone, activated via the RAA system, elevate blood pressure through vasoconstriction as well as sodium and water retention, respectively. Antidiuretic hormone further exacerbates fluid retention through V2 receptor-mediated water reabsorption, while also contributing to vasoconstriction and neurohormonal activation, thereby amplifying cardiorenal stress with the CKM syndrome.^10^ This volume expansion increases blood pressure, blood volume and afterload, which, in turn, stimulates cardiac remodeling through hypertrophy and fibrosis. These pathological processes impair both renal and cardiac function, reinforcing the interconnected relationship between the heart and kidneys.^11^ Therefore, special attention should be given to cardiovascular and renal comorbidities, as these conditions significantly impact health status and disease progression. This is particularly important when selecting therapies, as specific GLP-1-based therapies offer benefits that extend beyond glycemic management.

## GLP-1-based therapies: Mechanistic overview

Incretins are gastrointestinal peptides released in response to nutrient intake that potentiate insulin secretion from pancreatic beta cells and induce glucose uptake. The two primary incretins are GLP-1, secreted by L-cells, and GIP, secreted by K-cells. GLP-1 acts on GLP-1 receptors that are widely expressed in the pancreatic islets, gastrointestinal (GI) tract, central nervous system, heart and kidneys. GLP-1 decreases gastric emptying in the GI tract, prolongs nutrient absorption and reduces postprandial glucose excursions.^12^ In addition to enhancing insulin release from beta cells during hyperglycemia, GLP-1 also suppresses glucagon secretion from alpha cells and inhibits hepatic gluconeogenesis through the gut–pancreas–liver axis.^13^ A study measured overnight plasma concentrations of glucose, insulin and glucagon, as well as glucose turnover, after GLP-1 infusion and found that plasma glucose levels decreased significantly, despite increases in plasma insulin and decreases in plasma glucagon.^14^ Additionally, another study demonstrated that an infusion of physiological prandial GLP-1 resulted in a significant decrease in hepatic glucose production with no effect on overall glucose disposal.^15^

Within the CNS, GLP-1 receptors are widely expressed in regions associated with appetite and energy homeostasis, including the hypothalamus and brainstem. Activation of these receptors plays a critical role in suppressing food intake and promoting satiety; GLP-1 receptor activation has been shown to enhance satiety by strengthening functional connectivity between the nucleus tractus solitarius (NTS) in the brainstem, the hypothalamus and the thalamus.^16^ Both GLP-1 and GIP are glucose-dependent insulinotropic hormones. Although GLP-1 primarily suppresses glucagon secretion during hyperglycemia, GIP retains its capacity to stimulate glucagon release, particularly in response to hypoglycemia, thereby supporting counterregulatory mechanisms.^17,18^ Other non-pancreatic effects of GIP include increased lipogenesis, enhanced lipoprotein lipase activity and potential bone formation.^19^

The mechanisms of action of GLP-1 RAs indicate benefits for the cardiovascular, renal and metabolic aspects of the CRM syndrome. Within the CRM syndrome, MASLD has become the most common liver disease, characterized by intrahepatocyte lipid accumulation and insulin resistance, a pathogenesis shared with T2D.^20^ Insulin resistance is characterized by decreased glucose uptake in skeletal muscle, increased hepatic glucose production, increased lipolysis in adipose tissue and increased hepatic de novo lipogenesis.^21^ GLP-1 and GIP agonists address this pathogenesis by decreasing hepatic glucose production, increasing insulin secretion and reducing hepatic de novo lipogenesis.^22,23^ The glycemic management facilitated by GLP-1 agonists also shows downstream cardio-and renal-protective benefits by reducing oxidative stress, decreasing vascular and systemic inflammation and attenuating atherosclerotic plaque development.^24^ Furthermore, activation of the GLP-1 receptor appears to have direct renal-protective effects, suggesting a potential mechanism to mitigate glomerular hyperfiltration, a significant contributor to diabetic nephropathy and albuminuria.^25^

*Cardiovascular (CV) Protective Mechanisms*. Beyond their well-established effects on glycemic and metabolic effects, GLP-1 RAs provide a range of additional CV benefits that largely operate independently of glucose reduction.^26,27^ These medications have been shown to reduce systemic inflammation, as indicated by decreases in circulating biomarkers like high-sensitivity C-reactive protein and interleukin-6.^28,29^ At the vascular level, activating the GLP-1 receptor enhances endothelial function by increasing oxide availability and decreases arterial stiffness, both of which are early factors in atherosclerosis development.^29,30^ Emerging preclinical and clinical evidence also points to direct anti-atherosclerotic effects, such as stabilizing vulnerable plaques through less macrophage infiltration and lower expression of pro-inflammatory adhesion molecules in the arterial wall.^26,29^ Additionally, small but meaningful reductions in systolic blood pressure and atherogenic lipid levels – especially post-meal triglycerides – further improve CV risk.^26,27^ These mechanisms, taken together, help explain the notable reduction in major adverse cardiovascular events (MACE) seen in large trials, even in participants who only achieved modest improvements in HbA1c.^27,30–32^

*Renoprotective Mechanisms*. The renoprotective effects of GLP-1 RAs extend well beyond glycemic management.^33,34^ A key proposed mechanism involves modulating tubuloglomerular feedback, where GLP-1 receptor activation promotes natriuresis at the proximal tubule, thereby reducing sodium delivery to the macula densa and decreasing intraglomerular hyperfiltration that drives progressive nephron loss in diabetic kidney disease.^33,35^ Simultaneously, these agents seem to mitigate renal injury through anti-inflammatory and anti-fibrotic pathways, including suppression of transforming growth factor-beta (TGF-β) signaling and reduction of extracellular matrix building up within the glomerular and tubulointerstitial compartments.^34,35^ These effects, clinically, are evident in sustained reductions in albuminuria across multiple CVOTs – reductions that surpass what would be expected from HbA1c improvements and that last over long-term follow-up.^30,36^ The landmark FLOW trial provided the first dedicated evidence from a kidney outcomes trial, demonstrating that semaglutide lowered major kidney disease events by 24% and slowed estimated glomerular filtration rate (eGFR) decline in individuals with T2D and CKD.^36^

Glucose-independent mechanisms position GLP-1 RAs not only as antihyperglycemic treatments but also as agents with inherent organ-protective properties. This supports their early and widespread use in individuals with T2D and high-risk or established CV and kidney conditions. The synergistic action of GLP-1-based therapies enhances insulin sensitivity, promotes weight reduction by increasing satiety and improves hepatic lipid metabolism and insulin responsiveness. In summary, these therapeutic modalities provide a comprehensive approach to managing hyperglycemia, atherosclerosis, renal impairment and hepatic steatosis.

## Cardiovascular outcomes

### GLP-1 RAs

GLP-1 RAs have shown advantages beyond HbA1c and body weight reduction, including impacts on cardiovascular diseases (CVD), which are significant causes of morbidity and mortality in individuals with T2D. Given the close relationship between diabetes and CVD, and the concern that some therapies could increase cardiovascular risk, in 2008, the FDA mandated CVOTs for T2D treatments.^37^ Among the CVOTs, ELIXA (lixisenatide), EXSCEL (exenatide), LEADER (liraglutide), PIONEER-6 and SOUL (oral semaglutide), REWIND (dulaglutide), SUSTAIN-6 (injectable semaglutide), HARMONY (albiglutide; off the market) and AMPLITUDE-O (efpeglenatide; not yet approved in the US), six trials showed a statistically significant difference in reducing the risk of major adverse cardiovascular events (MACE) when compared to placebo.^38–46^ The 3-point MACE included cardiovascular death, nonfatal stroke or nonfatal myocardial infarction, while the 4-point MACE additionally included hospitalization for unstable angina.

### Albiglutide

Although the HARMONY trial (Albiglutide and Cardiovascular Outcomes in Patients with Type 2 Diabetes and Cardiovascular Disease) established a superiority of albiglutide over placebo in persons with T2D and CVD, the drug was discontinued in 2017 due to prescription limitations and is no longer available for clinical use.^45,47^

### Dulaglutide

The Researching Cardiovascular Events with a Weekly Incretin in Diabetes (REWIND) trial evaluated the cardiovascular benefits of dulaglutide in individuals with T2D, both with or without prior cardiovascular disease.^43^ Participants receiving dulaglutide had a significantly lower risk of MACE, specifically nonfatal stroke; all-cause mortality, myocardial infarction (fatal and nonfatal) and cardiovascular mortality did not differ compared to placebo.^43^

### Efpeglenatide

Efpeglenatide was reported to reduce the risk of 3-point MACE in individuals with T2D and cardiovascular or renal disease in the AMPLITUDE-O trial (Effect of Efpeglenatide on Cardiovascular Outcomes); however, it has not received FDA approval at the time of writing.^46^

### Exenatide

The Exenatide Study of Cardiovascular Event Lowering (EXSCEL) trial demonstrated that exenatide was similar to placebo in terms of cardiovascular benefits or adverse events in individuals with T2D with or without cardiovascular disease.^39^

### Liraglutide

In the Liraglutide Effect and Action in Diabetes: Evaluation of Cardiovascular Outcome Results (LEADER) trial, liraglutide was studied in persons with established cardiovascular disease or cardiovascular risk factors and demonstrated a reduced risk of the 3-point MACE.^40^ Furthermore, liraglutide provided protection against all-cause mortality, myocardial infarction and microvascular complications such as nephropathy. However, individuals given liraglutide experienced significantly more adverse events compared to those receiving a placebo, specifically confirmed hypoglycemia (p=0.02) and gastrointestinal adverse events (e.g. nausea, vomiting, diarrhea and abdominal discomfort).^40^

### Lixisenatide

The Evaluation of Lixisenatide in Acute Coronary Syndrome (ELIXA) trial primarily assessed the cardiovascular benefits of lixisenatide in people with T2D and a recent acute coronary event within the past 180 days.^38^ The study concluded that lixisenatide did not reduce the risk of the composite 4-point MACE, its individual components or all-cause mortality. Still, it significantly increased the risk of gastrointestinal adverse effects compared to placebo in participants with T2D and recent acute coronary syndrome.^38^

### Semaglutide

The Trial to Evaluate Cardiovascular and Other Long-term Outcomes with Semaglutide in Subjects with Type 2 Diabetes (SUSTAIN-6), PIONEER-6 (A Trial Investigating the Cardiovascular Safety of Oral Semaglutide in Subjects With Type 2 Diabetes) and the Semaglutide Cardiovascular Outcomes Trial in Patients With Type 2 Diabetes (SOUL) trial assessed the cardiovascular outcomes for semaglutide compared to placebo.^29,30,32^ The SUSTAIN-6 trial concluded that injectable semaglutide significantly reduced HbA1c levels and the risk of MACE events, specifically nonfatal stroke and nonfatal MI, in individuals with T2D.^44^ In the PIONEER-6 trial, oral semaglutide was similar to placebo in cardiovascular outcomes. The newly published SOUL trial included a larger population with a longer median follow-up of 49.5 months. The primary outcome occurred in 12% of the oral semaglutide group, compared to 13.8% of the placebo group, resulting in a 14% relative risk reduction in 3-point MACE.^42^ Together, these three trials suggest that semaglutide offers cardiovascular safety and reduces the occurrence of MACE, as shown in the SUSTAIN-6 and SOUL trials.

Several of these CVOTs provide strong evidence that GLP-1 RAs, including semaglutide, dulaglutide and liraglutide, improve glycemic management and reduce the risk of MACEs. These agents should be considered not only for glycemic management but also as part of a comprehensive strategy to lower cardiovascular morbidity and mortality in individuals with T2D who have established cardiovascular disease or are at high cardiovascular risk. However, individuals should be advised to monitor gastrointestinal adverse events, such as nausea, vomiting and diarrhea.

**Table 1:** Baseline characteristics of participants in cardiovascular outcome trials of GLP-1 and dual GLP-1/GIP receptor agonists^38–46,49^

Trial (Drug)	Population	Trial size	Baseline CVD (%)	Mean baseline HbA1c (%)	Mean age (years)	Mean diabetes duration (years)	Median follow-up (years)
HARMONY, 2018 (albiglutide)	Participants aged 40 years or older with type 2 diabetes and cardiovascular disease	9,463	100%	8.7	64	14	1.6
AMPLITUDE-O, 2021 (efpeglenatide)	Participants with type 2 diabetes and either established cardiovascular disease, or kidney disease plus at least one additional cardiovascular risk factor	4,076	90%	8.9	65	15	1.81
ELIXA, 2015 (lixisenatide)	Participants with type 2 diabetes and a recent heart attack or hospitalization for unstable chest pain within the previous 180 days	6,068	100%	7.7	60	9	2.1
LEADER, 2016 (liraglutide)	Participants aged 50 years or older with type 2 diabetes and established cardiovascular or chronic kidney disease, or aged 60 years or older with at least one cardiovascular risk factor	9,340	81%	8.7	64	13	3.8
EXSCEL, 2017 (exenatide)	Participants with type 2 diabetes with or without prior cardiovascular disease	14,752	73%	8	62	12	3.2
SUSTAIN-6, 2016 (injectable semaglutide)	Participants aged 50 years or older with type 2 diabetes and established cardiovascular disease, chronic kidney disease, or both, or aged 60 years or older with at least one cardiovascular risk factor	3,297	83%	8.7	65	14	2.1
PIONEER-6, 2019 (oral semaglutide)	Participants at high cardiovascular risk, defined as age 50 or older with cardiovascular or chronic kidney disease, or age 60 or older with cardiovascular risk factors only	3,183	85%	8.2	66	15	1.3
SOUL, 2025 (oral semaglutide)	Participants 50 years or older with type 2 diabetes and hemoglobin A1c 6.5% to 10%, with established atherosclerotic cardiovascular disease and/or chronic kidney disease	9,650	100%	8	66	15	4.1
REWIND, 2019 (dulaglutide)	Participants aged 50 years or older with type 2 diabetes and a prior cardiovascular event, aged 55 years or older with subclinical atherosclerosis or renal disease, or aged 60 years or older with at least two cardiovascular risk factors	9,901	31%	7.2	66	11	5.4
SURPASS-CVOT, 2025 Ongoing (tirzepatide)	Participants with type 2 diabetes and established atherosclerotic cardiovascular disease	13,299	100%	8.4	64	15	4.0

*CVD = cardiovascular disease; ELIXA = Lixisenatide in Patients with Type 2 Diabetes and Acute Coronary Syndrome; EXSCEL = Exenatide Study of Cardiovascular Event Lowering; GIP = glucose-dependent insulinotropic peptide; GLP-1 = glucagon-like peptide-1; HARMONY = Albiglutide and Cardiovascular Outcomes in Patients with Type 2 Diabetes and Cardiovascular Disease; LEADER = Liraglutide and Cardiovascular Outcomes in Type 2 Diabetes; REWIND = Researching Cardiovascular Events with a Weekly Incretin in Diabetes; SOUL = Semaglutide Cardiovascular Outcome; SURPASS-CVOT = Effect of Tirzepatide Versus Dulaglutide on Major Adverse Cardiovascular Events in Patients With Type 2 Diabetes; SUSTAIN-6 = Semaglutide in Subjects with Type 2 Diabetes.*

## GLP-1/GIP dual RA

### Tirzepatide

The Effect of Tirzepatide Versus Dulaglutide on Major Adverse Cardiovascular Events in Patients With Type 2 Diabetes (SURPASS-CVOT) trial compared the cardiovascular outcomes of tirzepatide to dulaglutide in individuals aged 40 years and older with T2D and atherosclerotic cardiovascular disease (ASCVD).^48,49^ In people with type 2 diabetes and ASCVD, tirzepatide was noninferior to dulaglutide for 3-point MACE, with tirzepatide outperforming dulaglutide in glycemic management, weight reduction, systolic blood pressure reduction and triglyceride level reduction.^49^ The occurrence of primary endpoint event was 12.2% in the tirzepatide group and 13.1% in the dulaglutide group, with an upper limit of the confidence interval less than 1.05, signifying significance in noninferiority, and the upper limit above 1.0, indicating non-significance in superiority.^49^

Please refer to *Tables 1 and 2* below for summaries of all CVOTs mentioned.^38–46,49^

## Heart failure with preserved ejection fraction

### Injectable semaglutide

The STEP-H FpEF (Semaglutide in Patients with Heart Failure with Preserved Ejection Fraction and Obesity) trial evaluated the efficacy of injectable semaglutide in enhancing symptoms and functional capacity in individuals with heart failure with preserved ejection fraction (HFpEF).^50^ This multicenter, randomized, double-blind, placebo-controlled study assigned 529 participants to receive either 2.4 mg of subcutaneous semaglutide or placebo once weekly for 52 weeks, followed by a 5-week follow-up period. The primary endpoints were alterations from baseline in the Kansas City Cardiomyopathy Questionnaire Clinical Summary Score (KCCQ-CSS), a patient-reported instrument that assesses the severity of symptoms and physical limitations associated with heart failure and body weight. The results, depicted in *Table 3*, were analyzed using two estimands: a treatment policy estimand, which represents the policy’s actual effectiveness, and a hypothetical trial product estimand, designed to evaluate efficacy under complete adherence.^50,51^ Semaglutide resulted in notable improvements in patient-reported outcomes based on KCCQ-CSS, and 65.9% of those in the semaglutide group achieved a 10% or greater body weight reduction at the end of the trial.^50^ These benefits were consistent across the treatment policy and the trial product estimands, demonstrating their effectiveness in both real-w orld use and in ideal adherence settings.

### Tirzepatide

In parallel, the SUMMIT (Study of Tirzepatide in Participants With Heart Failure With Preserved Ejection Fraction and Obesity) trial examined the effects of tirzepatide on individuals with obesity and heart failure with preserved left ventricular function.^51^ The results detailed in *Table 3* demonstrated that tirzepatide was linked to a reduced risk of cardiovascular death or worsening heart failure compared to placebo. Additionally, participants reported significant improvements in symptom relief and functional capacity, as assessed by the KCCQ-C SS. The results of this trial suggested that tirzepatide may do more than reduce weight in obesity-related HFpEF; in addition, this medication may favorably alter the clinical trajectory of the disease by reducing the risk of worsening heart-failure events.

**Table 2:** Outcomes of cardiovascular outcome trials of GLP-1 and dual GLP-1/GIP receptor agonists^38–46,49^

Trial (Drug)	Intervention	Primary endpoint	MACE (HR [95% CI])	Non-fatal MI (HR [95% CI])	Non-fatal stroke (HR [95% CI])	CV mortality (HR [95% CI])	All-cause mortality (HR [95% CI])	Hospitalization for heart failure (HR [95% CI])
HARMONY, 2018 (albiglutide)	30–50 mg weekly	3-point MACE	0.78 [0.68–0.90]	NR (fatal and nonfatal MI=0.75 [0.61–0.90])	NR (fatal and nonfatal stroke=0.86 [0.66–1.14])	0.93 [0.73–1.19]	0.95 [0.79–1.16]	0.71 [0.53–0.94]
AMPLITUDE-O, 2021 (efpeglenatide)	4 mg weekly 6 mg weekly	3-point MACE	0.73 [0.58–0.92]	0.78 [0.55–1.10]	0.80 [0.48–1.31]	0.72 [0.50–1.03]	0.78 [0.58–1.06]	0.61 [0.38–0.98]
ELIXA, 2015 (lixisenatide)	20 μg daily	4-point MACE	1.02 [0.89–1.17]	1.03 [0.87–1.22]	1.12 [0.79–1.58]	0.98 [0.78–1.22]	0.94 [0.78–1.13]	0.96 [0.75–1.23]
LEADER, 2016 (liraglutide)	1.8 mg daily	3-point MACE	0.87 [0.78–0.97]	0.88 [0.75–1.03]	0.89 [0.72–1.11]	0.78 [0.66–0.93]	0.85 [0.74–0.97]	0.87 [0.73–1.05]
EXSCEL, 2017 (exenatide)	2 mg weekly	3-point MACE	0.91 [0.83–1.00]	NR (fatal and nonfatal MI=0.97 [0.85–1.10])	NR (fatal and nonfatal stroke=0.85 [0.70–1.03])	0.88 [0.76–1.02]	0.86 [0.77–0.97]	0.94 [0.78–1.13]
SUSTAIN-6, 2016 (injectable semaglutide)	0.5 mg weekly 1.0 mg weekly	3-point MACE	0.74 [0.58–0.95]	0.74 [0.51–1.08]	0.61 [0.38–0.99]	0.98 [0.65–1.48]	1.05 [0.74–1.50]	0.86 [0.48–1.55]
PIONEER-6, 2019 (oral semaglutide)	14 mg daily	3-point MACE	0.79 [0.57–1.11]	1.18 [0.73–1.90]	0.74 [0.35–1.57]	0.49 [0.27–0.92]	0.51 [0.31–0.84]	1.11 [0.77–1.61]
SOUL, 2025 (oral semaglutide)	14 mg daily	3-point MACE	0.89 [0.71–1.11]	0.74 [0.61–0.89]	0.88 [0.70–1.11]	0.93 [0.80–1.09]	0.91 [0.80–1.02]	NR
REWIND, 2019 (dulaglutide)	1.5 mg weekly	3-point MACE	0.88 [0.79–0.99]	0.96 [0.79–1.16]	0.76 [0.61–0.95]	0.91 [0.78–1.06]	0.90 [0.80–1.02]	0.93 [0.77–1.12]
SURPASS-CVOT, 2025 (tirzepatide)	15 mg weekly	3-point MACE	0.92 [0.83 - 1.01]*	NR (fatal and nonfatal MI=0.86 [0.74–1.00])	NR (fatal and nonfatal stroke=0.91 [0.76–1.09])	0.89 [0.77–1.02]	0.84 [0.75–0.94]	0.96 [0.79–1.17]

*95.3% CI with p=0.003 for noninferiority and p=0.09 for superiority.

*AMPLITUDE-O = Effect of Efpeglenatide on Cardiovascular Outcomescheck Resolve; CI = confidence interval; CV = cardiovascular; ELIXA = Evaluation of Lixisenatide in Acute Coronary Syndrome; EXSCEL = Exenatide Study of Cardiovascular Event Lowering; GIP = glucose-dependent insulinotropic peptide; GLP-1 = glucagon-like peptide-1; HARMONY = Albiglutide and Cardiovascular Outcomes in Patients with Type 2 Diabetes and Cardiovascular Disease; HR = hazard ratio; LEADER = Liraglutide Effect and Action in Diabetes: Evaluation of Cardiovascular Outcome Results; MACE = major adverse cardiac events; MI = myocardial infarction; NR = Not reported in the original publication for that specific endpoint; PIONEER-6 = A Trial Investigating the Cardiovascular Safety of Oral Semaglutide in Subjects With Type 2 Diabetes; REWIND = Researching Cardiovascular Events with a Weekly Incretin in Diabetes; SOUL = Semaglutide Cardiovascular Outcome; SURPASS-CVOT = Effect of Tirzepatide Versus Dulaglutide on Major Adverse Cardiovascular Events in Patients With Type 2 Diabetes; SUSTAIN-6 = Trial to Evaluate Cardiovascular and Other Long-term Outcomes With Semaglutide in Subjects With Type 2 Diabetes.*

The 2026 ADA Standards of Care specifically recommend using a dual GIP/GLP-1 RA – tirzepatide – with proven benefit in individuals with symptomatic HFpEF and obesity.^52^

Both studies collectively emphasized the therapeutic potential of GLP-1-based agents for individuals with HFpEF and obesity. Semaglutide exhibited substantial benefits in weight loss and symptom relief, whereas tirzepatide showed enhancements in long-term cardiovascular outcomes and functional capacity. Considering the statistical significance and the degree of improvement across weight loss and symptom relief/functionality, semaglutide and tirzepatide may represent a valuable treatment option for alleviating the metabolic, functional and cardiovascular challenges commonly faced by those with HFpEF and obesity, many of whom also have diabetes.^50,51^

**Table 3:** Summary of STEP-HFpEF and SUMMIT trials in participants with heart failure with preserved ejection fraction^50,51^

Trial (drug)	Trial size	Baseline characteristics	Trial protocol	Results		Adverse effects
STEP-HFpEF, 2023 (semaglutide)	529	HFpEF	**Duration:** 52 weeks + 5-week follow-up **Intervention:** 2.4 mg subcutaneous semaglutide daily **Control:** Placebo	**Treatment policy estimand KCCQ-CSS score change:** Semaglutide: +16.6 points Placebo: +8.7 points [95% CI: 4.8 to 10.9] p<0.001 **Body weight (% change):** Semaglutide: -13.3% Placebo: -2.6% [95% CI, -11.9 to -9.4] p<0.001	**Trial product estimand KCCQ-CSS score change:** Semaglutide: +19.1 points Placebo: +10.3 points [95% CI: 5.9 to 11.7] **Body weight (% change):** Semaglutide: -15.1% Placebo: -2.4% [95% CI: -13.9 to -11.5]	Fewer serious AEs with semaglutide (13.3% versus 26.7%; p<0.001). Discontinuations due to mostly GI-related AEs were more common with semaglutide
SUMMIT, 2025 (tirzepatide)	731	Heart failure (NHYA Class II-IV), LVEF>50% and BMI >30	**Median follow-up duration:** 104 weeks **Intervention:** 15 mg subcutaneous tirzepatide weekly **Control:** Placebo	**Death from cardiovascular causes or worsening heart failure** Tirzepatide: 36 patients Placebo: 56 patients HR 0.62 [95% CI: 0.41 to 0.95], p=0.026 **Worsening heart failure:** HR 0.54 [95% CI: 0.34 to 0.85] **Worsening heart failure resulting in hospitalization:** HR 0.44 [95% CI: 0.22 to 0.87] **Death from any cause:** HR 1.25 [95% CI: 0.63 to 2.45] **Mean change in KCCQ-CSS score:** Tirzepatide: +19.5 points Placebo: +12.7 points [95% CI: 3.3 to 10.6], p<0.001		More discontinuations with tirzepatide due to AEs (6.3% versus 1.4%), primarily GI-related AEs (4.1% versus 0%)

*AEs = adverse events; BMI = body mass index; CI = confidence interval; GI = gastrointestinal; HFpEF = heart failure with preserved ejection fraction; HR = hazard ratio; KCCQ-CSS = Kansas City Cardiomyopathy Questionnaire Clinical Summary Score ; LVEF = left ventricular ejection fraction; NYHA = New York Heart Association; STEP-HFpEF = Semaglutide in Patients with Heart Failure with Preserved Ejection Fraction and Obesity; SUMMIT = Study of Tirzepatide in Participants With Heart Failure With Preserved Ejection Fraction and Obesity.*

**Table 4:** Summary of the FLOW trial: semaglutide versus placebo in patients with type 2 diabetes and chronic kidney disease^36^

Trial (Drug)	Trial size	Baseline characteristics	Trial protocol	Results	Adverse effects
FLOW, 2024 (semaglutide)	3,533	T2D and CKD	**Median follow-up period:** 3.4 years **Intervention:** 1.0 mg semaglutide weekly **Control:** Placebo	Composite primary outcomes* HR 0.76 [95% CI: 0.66 to 0.88], p=0.0003 NNT=20 [95% CI: 14 to 40] **Annual rate of eGFR decline:** Semaglutide: -2.19 mL/min/1.73 m²/year Placebo: -3.36 mL/min/1.73 m²/year [95% CI: 0.86 to 1.47], p<0.001	Fewer serious AEs with semaglutide (49.6% versus 53.8%) due to fewer infections and CV events. Slightly more discontinuations with semaglutide (233 [13.2%] versus 211 [11.9%])

*Composite primary outcome includes major renal events, defined as follows:

*– Onset of kidney failure requiring long-term dialysis, kidney transplantation, or sustained eGFR <15 mL/min/1.73 m² for >28 days.*

*– ≥50% decline in eGFR sustained for >28 days.*

*– Death from renal or cardiovascular causes.*

*AEs = adverse events; CI = confidence interval; CKD = chronic kidney disease; CV = cardiovascular; eGFR = estimated glomerular filtration rate; FLOW = Evaluate Renal Function with Semaglutide Once Weekly; HR = hazard ratio; NNT = number needed to treat; T2D = type 2 diabetes.*

## Renal outcomes

The recently published FLOW (Evaluate Renal Function with Semaglutide Once Weekly) trial, a multicenter, double-blind, randomized, placebo-controlled study, investigated whether injectable semaglutide reduces the risk of kidney failure, cardiovascular events and mortality in individuals with T2D and moderate-to-severe CKD, regardless of the presence of established ASCVD.^36^ *Table 4* summarizes the details of this trial.^36^ The FLOW trial provides compelling evidence that semaglutide significantly reduces the annual decline in eGFR and albuminuria in individuals with T2D and CKD, consistent with prior findings from secondary analyses of CVOTs. These positive renal outcomes add to the role of injectable semaglutide in improving glycemic management and reducing established ASCVD in individuals with T2D, thereby expanding therapeutic options for CKD in T2D beyond sodium-glucose cotransporter 2 (SGLT-2) inhibitors.

## Metabolic liver and weight management outcomes

According to the World Health Organization (WHO), obesity is defined as the abnormal or excessive accumulation of fat that presents a health risk, resulting from a combination of environmental factors, such as diet and physical inactivity, as well as genetic predisposition.^38^ In recent years, a substantial body of clinical evidence has demonstrated the efficacy of GLP-1 and dual GLP-1/GIP RAs as effective therapeutic modalities not only for glycemic management but also for the management of metabolic liver diseases and obesity.^53–57^ These landmark trials collectively signify a paradigm shift in the treatment of obesity and the potential treatment of metabolic liver diseases in individuals with or without type 2 diabetes. *Table 5* (metabolic liver disease) and *Table 6* (obesity) summarize key trial parameters, outcomes and clinical significance across these studies.^54–58^

**Table 5:** Summary of semaglutide clinical trials in metabolic–associated steatohepatitis^54–56^

Trial (Drug)	Trial Size	Baseline characteristics	Trial protocol	Results	Adverse effects
A placebo–controlled trial of subcutaneous semaglutide in nonalcoholic steatohepatitis, 2018 (semaglutide)	320	Biopsy-confirmed NASH with liver fibrosis stages F1 to F3	**Duration:** 72 weeks **Intervention:** 0.1 mg, 0.2 mg, or 0.4 mg subcutaneous semaglutide daily **Control:** Placebo	**Resolution of NASH with no worsening of fibrosis:** Semaglutide 0.1 mg: 40% Semaglutide 0.2 mg: 36% Semaglutide 0.4 mg: 59% (p<0.001) Placebo: 17% **Fibrosis stage improvement:** Semaglutide 0.4 mg: 43% Placebo: 33% p=0.48 **Mean per cent weight loss:** Semaglutide 0.4 mg: 13% Placebo: 1%	GI-related AEs were more common with semaglutide 0.4 mg (e.g. nausea 42% versus 11%; vomiting 15% versus 2%)
ESSENCE, 2025 (semaglutide)	1,197	Biopsy-confirmed MASH with liver fibrosis stage F2 or F3	**Duration:** 240 weeks **Intervention:** 2.4 mg subcutaneous semaglutide weekly **Control:** Placebo	**Resolution of steatohepatitis without worsening of fibrosis:** Semaglutide: 62.9% Placebo: 34.3% [95% CI: 21.1–36.2]; p<0.001 **Reduction in liver fibrosis without worsening of steatohepatitis:** Semaglutide: -36.8% Placebo: -22.4% [95% CI: 7.5–21.3]; p<0.001	GI-related AEs
Tirzepatide for metabolic dysfunction-associated steatohepatitis with liver fibrosis, 2024 (tirzepatide)	190	Biopsy-confirmed MASH with stage F2 or F3 fibrosis and NAFLD activity score >4	**Duration:** 52 weeks **Intervention:** 5 mg, 10 mg, or 15 mg subcutaneous tirzepatide weekly **Control:** Placebo	**Resolution of MASH with no worsening of fibrosis:** Tirzepatide 5 mg: 44% (p<0.001) Tirzepatide 10 mg: 34% (p<0.001) Tirzepatide 15 mg: 56% (p<0.001) Placebo: 10% **Reduction in liver fibrosis without worsening of steatohepatitis:** Tirzepatide 5 mg: 55% [95% CI: 5 to 46] Tirzepatide 10 mg: 25% [95% CI: 1 to 42] Tirzepatide 15 mg: 51% [95% CI: 1 to 42] Placebo: 30%	High overall AE rates with tirzepatide (92% versus 83%), mostly mild/moderate GI-related events. Discontinuation and serious AE rates were similar between tirzepatide and placebo (both 4% and 6%, respectively)

*AEs = adverse events; CI = confidence interval; ESSENCE = The Effect of Semaglutide in Subjects With Non-cirrhotic Non-alcoholic Steatohepatitis; GI = gastrointestinal; MASH = metabolic–associated steatohepatitis; NAFLD = non-alcoholic fatty liver disease; NASH = Non-alcoholic steatohepatitis.*

**Table 6:** Summary of key trials evaluating semaglutide and dulaglutide in weight management outcomes^57,58^

Trial (Drug)	Trial Size	Baseline characteristics	Trial protocol	Results	Adverse effects
STEP-1, 2021 (semaglutide)	1,950	**Mean body** **weight:** 105.3 kg **Mean BMI:** 37.9	**Duration:** 68 weeks **Intervention:** 2.4 mg SQ semaglutide weekly **Control:** placebo weekly	**Percent change in body weight:** Semaglutide: -14.9% Placebo: -2.4% [95% CI: -13.4 to -11.5]; p<0.001 **Achievement of ≥5% reduction of body weight:** Semaglutide: 86.4% (1,047 participants) Placebo: 31.5% (182 participants) p<0.001	GI-related AEs (*e.g. nausea and diarrhea*)
SUSTAIN-7, 2018 (semaglutide & dulaglutide)	1,201	**Mean Body** **Weight:** 95.2 kg **Mean BMI:** 33.5	**Duration:** 40 weeks Comparisons: Low Dose: Semaglutide 0.5 mg versus Dulaglutide 0.75 mg High Dose: Semaglutide 1 mg versus Dulaglutide 1.5 mg	**Mean body weight loss:** Low-dose comparison semaglutide 0.5 mg: -4.6 kg Dulaglutide 0.75 mg: -2.3 kg [95% CI: -4.32 to -2.78]; p<0.0001 High Dose Comparison Semaglutide 1 mg: -6.5 kg Dulaglutide 1.5 mg: -3.0 mg [95% CI: -4.32 to -2.78]; p<0.0001	GI-related AEs (*e.g. nausea, diarrhea, vomiting and anorexia*)

*AEs = adverse events; BMI = body mass index; CI = confidence interval; GI = gastrointestinal; SQ = subcutaneous; STEP-1 = Semaglutide in Patients; SUSTAIN-7 = Efficacy and Safety of Semaglutide Versus Dulaglutide as add-on to Metformin in Subjects With Type 2 Diabetes.*

## Safety considerations

The main tolerability issue with GLP-1-based therapies remains gastrointestinal side effects, especially nausea, vomiting, diarrhea, constipation and treatment discontinuation during dose escalation.^59^ A safety discussion should also include boxed warnings and contraindications related to medullary thyroid carcinoma/MEN2, as well as important precautions like pancreatitis, gallbladder disease and hypoglycemia when combined with insulin or sulfonylureas, hypersensitivity reactions, and, in some individuals, potential worsening of diabetic retinopathy with semaglutide.^60^ Slowing the dose titration, offering anticipatory counseling and carefully choosing concurrent therapies can help improve treatment adherence.

## Clinical integration and the 2026 ADA SOC clinical guidelines

The 2026 ADA SOC in Diabetes, guiding healthcare professionals in the prevention, management and treatment of the condition, now prioritizes treatment algorithms that focus on risk and complication management, particularly comorbidities related to the CRM syndrome, rather than solely emphasizing glucose management.^52^ These updated guidelines recognize the links among T2D, heart disease, kidney dysfunction and obesity and highlight the importance of integrating cardiovascular and renal comorbidities into therapeutic decision-making.

### Cardiovascular diseases

The GLP-1-based therapies are prominently featured in the ADA treatment algorithm for managing T2D. For people with established ASCVD or at high risk, the recommendation is to use a GLP-1 RA with proven CVD benefit or an SGLT2 inhibitor with proven CVD benefit. The GLP-1 RAs that have demonstrated benefit in randomized, controlled trials are liraglutide, semaglutide and dulaglutide.^40–44^

While SGLT2 inhibitors are recommended for people with heart failure, the guidelines do recommend using a GLP-1 RA that has demonstrated benefit for people with heart failure with preserved ejection fraction and obesity. Injectable semaglutide and tirzepatide have both shown benefits in this population.^50,51^

### Renal disease

New in 2025, GLP-1 RAs with proven benefits are also considered first-line options for people with chronic kidney disease (CKD). Similar to ASCVD, SGLT2 inhibitors or GLP-1 RAs with proven benefits are preferred. Although several GLP-1 agents have demonstrated positive kidney outcomes as secondary endpoints, only semaglutide showed improved kidney outcomes as a primary endpoint in the FLOW trial.^36^

### Metabolic conditions: MASLD and obesity

According to the 2026 ADA Standards of Care in Diabetes, in mitigating risk of MASLD or MASH, the use of a GLP-1 RA or a dual GLP-1/GIP RA with proven or potential benefit is recommended, or in conjunction with pioglitazone, for this population.^43^ The risk can be assessed using a Fib-4 score, and an imaging test, such as transient elastography, can subsequently confirm the presence of steatosis.

For those who do not need complication risk reduction, the choice of agent should depend on whether weight management is a goal. Approximately 90% of people with T2D live with overweight or obesity, so this is often a desired outcome.^53,61^ The ADA guidelines clearly state that the most efficacious agents for weight loss include semaglutide and tirzepatide, and highly efficacious agents include dulaglutide and liraglutide.^43^ Other GLP-1 RAs, like exenatide, are considered intermediate in efficacy for weight management.^43^

### Complementary role with SGLT-2 inhibitors and residual CRM risk

In the context of CRM, GLP-1-based therapies and SGLT-2 inhibitors should be considered complementary rather than competing options. The ADA 2026 Standards of Care in Diabetes continue to employ a risk-based framework and explicitly categorize SGLT-2 inhibitor and GLP-1 receptor agonist therapy within a broader guideline-based approach to reducing cardiovascular, renal and metabolic risk.^43^ Clinically, SGLT-2 inhibitors remain particularly advantageous when HF or CKD progression is the primary concern. Conversely, GLP-1 RAs and dual GIP/GLP-1 agonists are particularly appealing when obesity, ASCVD, HFpEF with obesity and MASH are prevalent. For selected patients with persistent residual CRM risk, combination therapy may be considered reasonable when it is well-tolerated and accessible.

## Health equity and implementation challenges

Despite the accumulating evidence supporting the efficacy of GLP-1 and dual GLP-1/GIP RAs in mitigating CRM risk, substantial disparities in access, prescribing and affordability persist as significant impediments to their widespread adoption, particularly among vulnerable populations that require these therapeutic interventions most urgently. Numerous studies have demonstrated that individuals belonging to racial and ethnic minority groups, low-income communities and those who are publicly insured or uninsured are frequently less likely to receive GLP-1-based therapies, even when clinical indications are evident.^62–64^ These disparities exacerbate the existing disparities in the burden of T2D, CVD, obesity and CKD, which disproportionately affect historically marginalized populations. Indeed, analyses of broader prescribing data have demonstrated that Black adults with diabetes are approximately 27% less likely than White adults to utilize newer diabetes drug classes; this disparity has progressively widened with each successive wave of drug approvals.^65–67^

Zhao et al. reported that among Medicare-insured adults with CKD, the initiation rates of SGLT-2 inhibitors and GLP-1 RAs were significantly lower among Black and Hispanic patients compared to White patients, even after accounting for clinical and sociodemographic factors.^62^ Similar patterns have been observed in broader analyses of GLP-1 RA prescribing. Kukhareva et al. discovered significant racial and ethnic disparities in prescription rates that persisted across payer types and geographic regions.^63^ Data from the Look AHEAD study further validated these findings, indicating that racial/ethnic minority participants and those with lower socioeconomic status were significantly less likely to receive newer diabetes medications, including GLP-1 RAs.^64^

Multiple structural barriers contribute to these disparities. Prior authorization (PA) requirements impose a significant administrative burden on prescribers, delaying or preventing the initiation of guideline-recommended therapies. Recent data indicate that PA requirements for GLP-1 RAs in Medicare Part D plans increased from below 15% in the third quarter of 2023 to over 80% by the third quarter of 2024.^68^ High out-of-pocket expenses remain a significant barrier for many patients. Branded GLP-1 RAs often have list prices exceeding $1,000 per month, making them unaffordable without sufficient insurance coverage; consequently, the average monthly out-of-pocket costs for certain agents have nearly doubled between 2024 and 2025.^69^ The majority of state fee-for-service Medicaid programs either exclude GLP-1 RAs for weight management or impose PA criteria more restrictive than the FDA labelling.^70,71^ Beyond insurance and prescriber inertia—particularly among primary care providers who manage the majority of people with T2D—underutilization is driven by clinicians’ unfamiliarity with the cardiorenal benefits of these agents or overestimating patients’ reluctance to injectable therapies.^71,72^ Low health literacy significantly limits patient engagement, leading to insufficient understanding of coverage options, treatment benefits and the paramount importance of medication adherence.

Addressing these implementation gaps will require coordinated, multi-level strategies. At the health-system level, integrating clinical decision support tools, with the help of artificial intelligence, into electronic health records can incentivize clinicians to consider GLP-1 or dual RA for eligible individuals with T2DM and established cardiovascular or renal disease. This approach can mitigate prescriber inertia and ensure that care aligns with established guidelines. Community health worker (CHW) programs have demonstrated efficacy in enhancing medication adherence and chronic disease outcomes among underserved populations. Systematic reviews of CHW-led interventions among Latino and African American adults with T2D have reported substantial reductions in A1c levels and improved medication adherence when integrated into standard care.^73^ These programs present a promising model for supporting patients initiating injectable therapies. To address cost-related obstacles, it is imperative to expand the utilization of manufacturer patient assistance programs, advocate for the inclusion of formulary medications within Medicaid and Medicare Part D plans and implement legislative measures to limit out-of-pocket expenses for cardiometabolic medications. Telehealth platforms can facilitate dose titration and follow-up monitoring, particularly for individuals in rural or medically underserved areas who encounter geographic barriers to specialist care. Emerging real-world evidence suggests that telehealth-managed GLP-1 RA treatment can achieve weight loss outcomes comparable to those observed in clinical trials.^74^

## Future directions

The therapeutic landscape for GLP-1-based therapies continues to evolve far beyond the original focus on glucose management, with several high-impact trials currently underway. The SURPASS-CVOT directly compares tirzepatide and dulaglutide in individuals with T2D and high risk for CVD.^48^ The results, expected in 2025, could clarify whether tirzepatide offers ASCVD protection, potentially serving as a new option for mitigating the CRM risk in T2D.

In addition to dual agonism, a GLP-1/GIP/glucagon triple receptor agonist, retatrutide, has shown promising results, inducing substantial weight loss and lowering HbA1c levels in clinical trials.^75,76^ The TRIUMPH OUTCOMES trial assesses the cardiovascular safety of retatrutide, with both cardiovascular and renal endpoints as primary outcomes.^77^

Ongoing trials in substance use disorders, including alcohol, smoking, cocaine, cannabis and narcotics, are being conducted to determine the potential efficacy of the GLP-1-based therapies in managing these conditions due to the links between the reward system and the pathophysiology of such conditions.^78^ In addition, these therapies have also been studied, specifically exenatide, in people with Parkinson’s disease. Positive outcomes on stopping/delaying disease progression have been reported in trials involving exenatide twice daily, exenatide once weekly and lixisenatide once daily.^79–81^ The results from these trials have set a strong foundation for further investigation. Given their roles in managing chronic conditions, the potential benefits of GLP-1-based therapies will continue to be explored, and their positive outcomes may benefit those living with multiple chronic conditions.

## Summary and conclusions

Over the past decade, the management of type 2 diabetes has undergone a transformation, shifting its primary focus from glucose management to a comprehensive approach that addresses cardio–renal–metabolic risk reduction. A substantial body of evidence derived from cardiovascular and renal outcome trials has demonstrated that GLP-1 receptor agonists and, more recently, GLP-1/GIP dual agonists represent transformative therapies that extend their benefits beyond a glucose-centric approach. These agents have consistently demonstrated reductions in major adverse cardiovascular events, slowed the progression of chronic kidney disease and produced significant improvements in weight and liver outcomes for individuals with or without type two diabetes. These findings have continually shaped the ADA Standards of Care in Diabetes, and the 2025 edition recommends using these therapies regardless of baseline HbA1c in individuals at high risk of CRM.

The timely and equitable adoption of GLP-1-based therapies can substantially enhance the standard of care for type 2 diabetes by addressing the entire spectrum of CRM syndrome. To fully realize the potential of these agents, efforts must prioritize equitable access, early intervention and integrated chronic disease management strategies that align with a CRM-focused approach. GLP-1-based therapies currently intersect type 2 diabetes with obesity, cardiovascular disease, kidney disease and metabolic liver conditions, marking a paradigm shift in the prevention and treatment of complex chronic diseases.
